# On the Molecular Theory of Organization

**Published:** 1861

**Authors:** J. Hughes Bennett

**Affiliations:** Professor of the Institutes of Medicine, and Senior Professor of Clinical Medicine in the University of Edinburgh


### ON THE MOLECULAR THEORY OF ORGANIZATION

By J. HUGHES BENNETT, M.D., F. R. S. E.,
*Professor of the Institutes of Medicine, and Senior Professor of Clinical Medicine in the University of Edinburgh.*

Parodying the celebrated expression of Harvey—viz: "Omne animal ex ovo," it has been attempted to formularize the law of development by the expression, "Omnis cellula e cellula," and to maintain "that we must not transfer the seat of real action to any point beyond the cell."* In the attempts which have been made to support this exclusive doctrine, and to give all the tissues and all the vital properties a cell origin, the great importance of the molecular element, it seems to me, has been strangely overlooked. It becomes important, therefore, to show that real action, both physical and vital, may be seated in minute particles, or molecules much smaller than cells, and that we must obtain a knowledge of such action in these molecules if we desire to comprehend the laws of organization. To this end I beg to direct attention—

Ist. To a description of the nature and mode of origin of organic molecules.

2nd. To a demonstration of the fact that these molecules possess inherent powers of forces, and are present in all those tissues which manifest vital force.

3rd. To a law which governs the combination, management, and behavior of these molecules during the development of organized tissue.

I. By a *molecule* is to be understood a minute body, seen under high magnifying powers in all organic fluids and textures, varying in size from the four thousandth of an inch down to a scarcely visible point, which may be calculated at much less than the twenty thousandth of an inch in diameter. Optically it is distinguished according to its size, the smallest presenting dark or light points as the focus is changed, and the larger exhibiting a dark or light centre, surrounded by a distinctly-shadowed ring. These last are frequently distinguished by the name of *granules*. The ultimate molecule

* Virchow, English Translation, p. 3.

has never been reached even with the highest magnifying powers. In the same manner that the astronomer with his telescope revolves nebulæ into clusters of stars and sees other nebulæ beyond them, so the histologist with his microscope magnifies molecules into granules and sees further molecules come into view. The chemical composition of these molecules must vary infinitely, but I have been in the habit of classifying them into three groups, and referring them to (1st) the albuminous, (2nd) the fatty, and (3rd) the mineral compounds. These constituents may be mingled together in various proportions so as to produce simple and compound molecules. In the vast majority of cases they are globular in shape, but they may be angular, square, and of various forms. They may differ in size or be of tolerably uniform size in the same liquid or substance. They may be regularly or irregularly diffused in the matter examined. Sometimes they are concentrated in particular places, and at others scattered in groups. Their color is various. Most of the pigments in plants and animals are dependent on the formation of molecules, which in the human lung have been proved to be pure carbon, and in the tissues of plants and animals differently tinted kinds of fat or of wax.

These molecules may be formed in two different ways: 1st, by precipitation in fluids; 2nd, by the disintegration of previously formed tissues. The former may be called *histogenetic* and the latter *histolytic*. They may be denominated molecules of formation and molecules of disintegration.

*Histogenetic molecules* are formed either from the union of two simple organic fluids, or from precipitations occurring in formative fluids, holding various substances in solution. Fourteen years ago I read to the Royal Society of Edinburgh a paper giving an account of the results obtained by a union of oil and liquid albumen, the two organic fluids from which molecular matter is most commonly derived. It was Dr. Ascherson, of Berlin, who first discovered the important fact, that the mere contact of oil and fluid albumen caused the latter to coagulate in the form of a membrane, which he called the haptogen membrane, from *aptomai*, to come in contact. A more complete mixture of two such drops produces, as is well known, a white opaque fluid or emulsion, which in structure exactly resembles milk. That is to say, surrounded by a layer of membrane of coagulated albumen. Such compound molecules possessing the property of endosmose may therefore readily be produced artificially, and by trituration can be reduced in size so as to resemble the elementary molecules in chyle or in the yolk of an egg. If oil and albumen be introduced into the stomach and intestinal canal, they are always so reduced; and one of the objects of digestion would appear to be, separating from the food and rendering fluid its oil and albumen, so as to produce the chylemolecules which are ultimately transformed into blood. Indeed, everywhere in living organisms it may be observed that oil and albumen, formed as secretions by plants, and entering the bodies of animals as food, either separately or united, constitute the chief origin of molecular formations.

Mr. Rainey has recently pointed out the condition which causes molecular mineral matter to assume the form of rounded nuclear bodies.* This condition is viscosity. If carbonate of lime be dissolved in water, the forms produced on its precipitation are crystalline; but if the fluid be glutinous—composed, for example, of fluid, gelatine or gum—the forms produced are oval or globular. Precipitations made in this way on slides of glass, closely resemble the appearances called nuclear or cellular in different stages of development. Mr. Rainey has further shown how starch granules are produced in the juices of vegetables by the endosmose of gum into a cell containing a solution of dextrine.† In the same manner that the contact of oil and albumen produces oleo-albuminous molecules, so does the contact of gum and dextrine precipitate starch molecules. In this manner we can comprehend how the mixture of various organic fluids gives rise to particles of different kinds.

*Histolytic molecules* are the result of the transformation and disintegration of fluid and solid substances by chemical and or mechanical action. They are generally larger in size than histogenetic molecules, are more purely fatty, and, from being associated with the *débris* of broken-down texture, may in most instances be readily distinguished. Thus in the breaking up of cells and of muscles when they become fatty, or in the putrefaction of vegetable or animal matters, these may be seen to soften, lose their peculiar structure, break up, and ultimately be reduced to a molecular condition.

We shall subsequently see that these two kinds of mole-

* On the Mode of Formation of Shells of Animals, of Bone, and of several other structures, by a process of Molecular Coalescence, &c. By Geo. Rainey, M. R. C. S. London, 1855.

† Microscopical Journal, 1859.

cules are constantly changing places; or, in other words, molecular matter formed from the process of integration may, when placed under peculiar circumstances, become the basis of matter which undergoes development. In nature, the breaking down of one substance is the necessary step to the formation of another; and the hystolytic or disintegrative molecules of one period become the histogenetic or formative molecules of another. The fact constitutes the basis of the law which I shall subsequently seek to establish.

II. These molecules are governed by forces, which induce amongst them a variety of movements, and cause them to combine in definite ways. This force, which we may call *molecular force*, is altogether independent of cell, nucleus, or other form of structure.

1st. There are the well known molecular movements described by Robert Brown. These vibratile, circular, serpentine, or irregular motions may be observed whenever molecules are suspended in fluids of certain desitines, but are too well known to require notice here. They occur altogether independent of organized structures, and must be regarded as in their nature purely physical.

2nd. The peculiar movements observed in the interior of cells, vegetables or animal, and during the putrefaction of organic matter. The former are seen in the large vegetable cells of the Chara, Valisneria, and Tradescantia, amongst plants; and those of chyle, the yelk of the egg, and of the salivary cell amongst animals. I have frequently watched the formation of the latter in putrid fluids. A scum, composed of molecules, collects on the surface; gradually several of these unite in minute filaments, more or less long, which assume vibratile or serpentine movements. They are then called *vibriones.* It has been much disputed whether this class of molecular motions be physical or vital.

3rd. The movements, which are unquestionably vital, that occur in the molecules of the yelk, on the entrance into the ovum of the spermatozoid. Here it cannot be maintained that the results are purely physical, because in different ova we see such widely varying effects from apparently the same cause. Neither can it be attributed to any direct influence of the cell or of its nucleus—the germinal vesicle. For example, an egg is fully matured in the female organs of generation, and would prove abortive if a spermatozoid did not find its way through the zona-pellucida and get amongst the molecules of the yelk. As soon as it does so, the apparently purposeless Brunonian movements receive a new impulse and direction. Both spermatozoid and germinal vesicle are dissolved amongst them; and that wonderful phenomenon of the division of the yelk takes place, not by cleavage or other action of the cell-wall or nucleus, but by the separation of the mass into two masses instead of one. This may be compared to what is observable in a dense crowd of men called upon to pass over to the right or left hand in order to settle any disputed question by majority. At first unusual confusion is communicated to the whole; some hurry in one direction, others in another; but, after a time, there is seen at the margins, where the crowd is less dense, a clear space, which gradually approaches the centre, and at length bisecting the whole, produces a complete segregation of the crowd into two portions. So with the molecules of the yelk in the egg after impregnation; their movements are directed by conditions which did not previously exist, and a stimulus is imparted to them which causes the peculiar result. It is the division and subdivision of the yelk, wholly or in part, which produced the germinal mass out of which the embryo is formed, and this not by any direct influence of the cell or nucleus, but in consequence of a power inherent in the molecules themselves, which is communicated to them for a specific purpose.

4th. The peculiar movements so well described by Brucke, Von Wittch, Harless, and especially by Lister, in the pigment-cells of the frog's skin;* and which occasion the sudden change of color in the chameleon, in fishes, and numerous other animals. The black pigment molecules may be diffused throughout the cell or concentrated in a mass, and all kinds of intermediate gradations may exist between diffusion and concentration. The change in color is owing to these alterations in the molecules, the tint being light when they are concentrated, and dark when they are diffused. Mr. Lister ascertained by experiment that their concentration is caused by exposure to light, by death of the animal, and by sudden section of the nerve going to the skin; while darkness and irritation of the nerve or skin causes diffusion. Sudden amputation of the limb produced at first diffusion, followed by the concentration of death. These movements of the pigment molecules are peculiarly vital, and altogether indepen-

* On the Cutaneous Pigmentary System of the Frog.—Philosophical Transactions, 1858

dent of the cell-wall or nucleus. The cell-wall is stationary, and acts only as a sac or investing membrane around the moving particles, while the concentrations of these about the nucleus is purely accidental, and frequently occurs in other parts of the cell. I have seen these molecules myself, as Mr. Lister describes them, streaming out to, and returning from, the circumference, under the influence of the stimuli referred to, where no cell nor nuclear action could be thought of.

5th. There are many other kinds of movements which are evidently independent of cells—for example, those of cilia and of spermatozoids. The former are outside cells, and the latter only move when they are liberated from the cells. The contractile fibrillæ of the muscle are evidently not dependent for their inherent powers on cells or other form of structure, but on the square-shaped molecules of which its substance is composed. All these phenomena, therefore, are connected with the molecules themselves; the force occasioning them is a molecular force, and has nothing to do with pre-existing cells, or supposed germinal centres, as some have imagined.

Again, the power of combination between these molecules, which, under peculiar conditions, not only move, but so move as to advance towards and press upon each other, that they at length unite and produce higher forms, must also be attributed to a molecular force operating in obedience to fixed laws. Thus it was demonstrated by Newton, that in a sphere the total attraction resulting from the particular attrantion of all its competent parts is, as regards any body drawn towards it, the same as if they had been concentrated at the centre. Hence minute spherical particles, as so many gravitating points, will be drawn towards each other with a force varying inversely as the squares of the distances between their respective centres. I may here refer to the able descriptions of Mr. Rainey,* as to the physical laws regulating the formation and disintegration of bodies by molecular attraction and repulsion as well as to the effects of molecular superposition, showing that the same physical power which leads to the formation of these artificial bodies, when long continued, causes their disintegration and destruction. All these changes occur slowly, and require time; but their contemplation, when regarded as purely physical phenomena, must strike us with surprise, as being closely allied to all our conceptions of the progress of life itself.

* *Op. cit.* See also papers in the Microscopical Journal, 1860.

In making use of the expression *life* and *vital action*, I am only using terms to indicate phenomena which, in the present state of science, cannot be accounted for by the ordinary laws of physics. Or it might be said that certain actions are directed and governed by conditions which are as yet undetermined, but which, as they only occur in organic, as distinguished from inorganic bodies, constitute vital actions. Not that an organized body is independent of physical forces, but that certain directions are communicated to them, which, as invariably resulting in specific forms or properties, make up the sum of what we call vitality.

Hence, although we see molecules combining in the forms of crystals and nucleated sperules, inasmuch as we have discovered the physical conditions on which they depend, and can produce them artificially, we have no difficulty in classifying these amongst purely physical phenomena, even when they occur in the interior of animals. But when other molecules unite to form nuclei, cells, and fibres, and these arrange themselves into tissues and organs to produce plants and animals, we are ignorant of the conditions by which these results are brought about—we cannot imitate them artificially, and are content to cal them vital. But the fact I am anxious to point out is this, that so far as observations and research have enabled us to investigate this difficult matter, it appears that the formations and disintegration of vegetables and animals, as well as the peculiar properties they exhibit, are essentially connected with the molecular element. Thus, when we investigate the functions of plants and animals—for example, generation, nutrition, secretion, metion, and sensation—we find them all necessarily dependent on the permanent existence and constant formation of molecules.

Thus generation, both in plants and animals, is accomplished by the union of certain molecular particles called the male and female elements of reproduction. Amongst the Protophyta, the conjugation of two cells enables their contents, or the endochrome, to mix together. This endochrome is a mass of colored molecules, and the union of two such masses constitutes the essential part of the generative act. In the Cryptogamia, a vibratile antheroid particle enters a germ-cell, and finds this last filled with a mass of molecules, which, on receiving the stimulus it imparts, assumes the power of growth. It is the same amongst the Phanerogamia when the germ-cell is impregnated by the pollen tube. In all these cases it is necessary to remember that the protoplasm is a mass of molecules; that a spore is another mass of molecules; that sporules are molecules; that antherozoids are only molecules with vibratile appendages; and that the so-called germinal matter of the ovule is also nothing but a mass of molecules. Cell-forms are subsequent processes, and once produced may multiply endogenously, by germination or cleavage; all that is here contended for is, that the primary form is molecular, and that the force producing action in it is a molecular force.

In animals, as in vegetables, every primary act of generation is brought about by the agency of molecules. The Protozoa entirely consist of mere molecular gelatiniform masses, in which it has never been pretended that a cell-wall or central cell exists. And yet such masses have the power of independent motion, and of multiplying by gemmation. Considerable discussion has occurred as to whether, amongst Infusorians, there is a union of sexes or a conjugation similar to what occurs amongst the Protophyta; but in either case it is by molecular fusion that the end is accomplished. In the higher classes of animals there are male elements, consisting of molecules, generally with, but sometimes destitute of, vibratile filaments; and female elements, composed of the yelk within the ovum, containing a germinal vesicle or included cell. Both spermatozoid and germinal vesicle are dissolved in the molecules of the yelk, which then, either wholly or in part, by successive divisions and transformations, constitute a germinal mass out of which the embryo is formed. Here, as in plants, it is necessary to remember that the spermatozoids, the yelk, and the germinal mass are all composed of molecules, and that these, combining together, form the nuclei, cells, fibres, and membranes which build up the tissues and organs of the organism. It is not from either the male or the female element that the embryo is formed. The supporters of an exclusive cell doctrine have endeavored to show that there is always a direct descent either from the wall of the ovum or from the germinal vesicle as its nucleus. Thus some consider that the vitelline membrane sends in partitions to divide the yelk mechanically. Others have formed the idea that the germinal visicle bursts, and that its included granules constitute the germs of those cells which subsequently form in the germinal mass. Others, again, suppose that on impregnation the germinal vesicle divides first, and that the molecules of the yelk are attracted round the two centres so formed. But numerous observations have satisfied me that both spermatozoid and germinal vesicle are simply dissolved amongst the molecules of the yelk, from the substance of which, stimulated and modified by the mixture so occasioned, the embryo is formed; a view which has further the merit of explaining what is known of the qualities of both parents observable in the offspring. I am only acquainted with one exception to this general law—namely, the development of *Pyrozoma*, recently described by Mr. Huxley, the description of which, however, is incomplete.* The truth appears to be, that in an analogous manner to that in which the pigment molecules of the skin are stimulated by the access of light to enter into certain vital combinations with one another, so are molecules of the yelk stimulated by the access of the spermatozoid to produce those other vital combinations that result in a new being. The essential action is not so much connected, as has hitherto been supposed, with the cell-wall or nucleus, as with the molecular element of the ovum.

With regard to nutrition—food and all assimilable material must be reduced in the first instance to the molecular form, while the fluid from which the blood is prepared—chyle—is essentially molecular. Most of the secretions originate in the effusion of a fluid into the grand-follicle, which becomes molecular and gives rise to cell formation. In muscle, the power of contractility is inherently associated with the ultimate molecules of which the fasciclus is composed; and, lastly, the gray matter of the sensory, ganglia and of the brain, which furnishes the conditions necessary for the exercise of secretion, and of even intellect itself, is associated with layers of molecules, which are unquestionably active in producing the various modifications of nervous force. These molecules are constant and permanent as an integral part of these tissues, as much as cells and fibres are essential parts of others, and their function is not transitory, but essential to the organs to which they belong.

All these facts point to the conclusion that vital action, so far from being exclusively seated in cells, is also intimately associated with the elementary molecules of the organism.

III. This leads me, in the third place, to an enunciation of the molecular law of growth, which a study of the numerous facts previously referred to has induced me to frame—viz: *That the development and growth of organic tissues are primarily owing to the successive formation of histogenetic and histolytic molecules.* We have already seen that development

* Annals of Natural History, Jan. 1860, p. 35.

and growth in animals originate in the molecules of the yelk of the egg, or of a germinal molecular mass formed from it. From numerous careful researches recognized by scientific men as giving a correct account of the development of various animals and textures, it would appear that the first form is molecular; that the moleculss unite to produce nuclei and cells; that these become disintegrated to produce a secondary mass of molecules; that these again unite to form secondary nuclei and cells; and that the same process is repeated more or less often in various developments, until the animal or tissue is formed. This constitutes the successive histogenetic and histolytic molecules observable in the process of growth —the former building up, to a certain extent, and the product disintegrating to produce the latter, which after a time, again, rearranges itself and becomes histogenetic to form cells or tissues, which in their turn break down and become histolytic. In short, not only development, but growth and secretion, absorption and excretion, are only different names given to histogenetic and histolytic processes, and these are brought about by formative and disintegrated molecules. As illustrations of this law, I may refer to the development of *Ascarix mistax*, as described by Nelson,* and of the process of nutrition in the human body.

In this, and a vast number of similar observations, it must be evident that a certain series of molecular transformations is necessary for the one which follows it. Thereby is produced a continual elaboration of matter,—a constant chemical and morphological series of changes,—the exact number and order of which, in the production of organic forms, only require time and perseverance to discover. Doubtless various conditions, dynamical, chemical and vital, must co-operate in producing the result, and they must all influence molecular as well as every other kind of combination. Such considerations and facts must convince us of the error of endeavoring to place the source of special vital action in any particular form or arrangement of organic matter, whether fibre, cell, nucleus, or molecule. Each and all of these elements have their vital endowments, which re-operate on the others. But, inasmuch as the molecular element is the first as well as the last form which organized matter assumes, it must constitute the principal foundation of organization itself.

It is not my object, in directing attention to a molecular

* Philosophical Transactions, 1850, plates xxviii, xxix, figs. 50, 68, 70, 78.

theory of organization, to interfere in any way with the well observed facts on which physiologists have based what has been called the cell-theory of growth. True, this last will require modification, in so far as unknown processes of growth have been hypothetically ascribed to the direct metamorphosis of cell-elements. But a cell once formed may produce other cells by buds, by division, or by proliferation, without a new act of generation, in the same manner that many plants and animals do; and this fact comprehends most of the admitted observations having reference to the cell doctrine. The molecular, therefore, is no way opposed to a true cell-theory of growth, but constitutes a wider generalization and a broader basis for its operations. Neither does it give any countenance to the doctrine of equivocal or spontaneous generation. It is not a fortuitous concourse of molecules that can give rise to a plant or animal, but only such a molecular mass as descends from parents, and receives the appropriate stimulus to act in certain directions.

In conclusion, the theory I have endeavored to establish on histological and physiological grounds is fully supported by all the known facts of disease and of morbid growths, which further serve to show that pathology, so far from being cellular, is, in truth, molecular.—*London Lancet.*

